# The impact of statin use before intensive care unit admission on patients with acute kidney injury after cardiac surgery

**DOI:** 10.3389/fphar.2023.1259828

**Published:** 2023-09-14

**Authors:** Shishi Li, Youlin Zhang, Yan Yang, Sining Chen, Zhiqian Yang, Chaoying Kuang, Yuzhen Zhong, Fanna Liu

**Affiliations:** ^1^ Department of Nephrology, The First Affiliated Hospital of Jinan University, Jinan University, Guangzhou, China; ^2^ Department of the Second Clinical, Shaanxi Provincial People’s Hospital, Xi’an, China; ^3^ School of Chemistry, Sun Yat-Sen University, Guangzhou, China

**Keywords:** CSA-AKI, statins, MIMIC-IV, prognosis, PSM

## Abstract

**Background:** Cardiac surgery-associated acute kidney injury (CSA-AKI) is a common and serious complication after cardiac surgery. The influence of statin use before surgery on the renal outcome of patients undergoing cardiac surgery is controversial. The purpose of this study was to evaluate the effect of statins on postoperative renal outcomes in patients undergoing cardiac surgery.

**Methods:** We included CSA-AKI patients in the Medical Information Mart for Intensive Care (MIMIC)—IV database and were divided into statin group and non-statin group according to whether they used statins before entering intensive care units (ICU). The main outcomes were hospitalization and 30-day mortality, and the secondary outcomes were 60-day mortality and 90-day mortality. We used propensity score matching (PSM) to adjust for confounding factors. The 95% confidence interval (CI) and risk ratio (RO) were calculated by the COX proportional regression model. At the same time, stratified analysis was used to explore whether the relationship between the statins use before intensive care units and mortality was different in each subgroup and whether the relationship between different doses of Atorvastatin and mortality was different.

**Result:** We identified 675 pre-ICU statin users and 2095 non-statin users. In the COX proportional regression model, pre-ICU statin use was associated with decreased in-hospital (HR = 0.407, 95%confidence interval 0.278–0.595, *p* < 0.001) and 30-day mortality (HR = 0.407, 95%CI 0.279–0.595, *p* < 0.001). The survival rate of patients who took statins before entering ICU was significantly higher than that of those who did not use statins at 30 days, 60 days and 90 days. There is a significant interaction between patients with aged>65 years (HR = 0.373, 95%CI 0.240–0.581, *p* < 0.001), Acute kidney injury grade I (HR = 0.244, 95%CI 0.118–0.428, *p* < 0.001), and without post-myocardial infarction syndrome (HR = 0.344, 95%CI 0.218–0.542, *p* < 0.001). The mortality in hospital and 60 days of CSA-AKI patients treated with ≥80 mg Atorvastatin before operation was significantly reduced (*p* < 0.05).

**Conclusion:** The pre-ICU statin use was significantly associated with decreased risk in hospital and 30-day mortality. The preoperative use of ≥80 mg Atorvastatin may improve the prognosis of CSA-AKI.

## 1 Introduction

Acute kidney injury (AKI), the sudden loss of kidney function, was classified according to the Kidney Disease Improving Global Outcomes (KDIGO) criteria, using serum creatinine and urine output (Ostermann et al., 2020; Kellum et al., 2021). AKI is a worldwide public health problem with a relatively high morbidity and death rate affecting millions of people, especially in intensive care units (ICU) (Bao et al., 2018; Zou et al., 2022). AKI is associated with earlier stages of chronic kidney disease (CKD), end-stage renal disease (ESRD), short-term morbidity, dialysis and mortality, and it usually complicates severe disease (Teo and Endre, 2017). Renal tubular ischemia is the main pathophysiological process of AKI, caused by major surgery, sepsis, hypovolemia, low cardiac output and medication toxicity (Matsa et al., 2014). It was reported that the incidence of cardiac surgery-associated AKI (CSA-AKI), the second most common cause of AKI, varies from 5% to 42% (Hobson et al., 2009). CSA-AKI was defined as patients who underwent cardiac surgery and met KDIGO criteria within 1 week of surgery (Wang and Bellomo, 2017). CSA-AKI not only increases the cost of care but also prolongs hospitalization and has 3–8 times perioperative mortality (Chertow et al., 1998; Metnitz et al., 2002). The pathophysiology of CSA-AKI may involve many pathways, including inflammation, oxidative stress, hypoperfusion, ischemia–reperfusion injury, mechanical factors, neuro-humoral activation and nephrotoxins (Wang and Bellomo, 2017). The treatment challenges for CSA-AKI were raised, and currently, the generally supportive measures are only available (Ostermann et al., 2021).

However, we can reduce the mortality of CSA-AKI through various management, such as early prevention measures. It is common for cardiac surgery patients to receive statins as part of their treatment. Statins stabilize atherosclerotic plaques, increase nitric oxide bioavailability, inhibit inflammatory responses and improve endothelial dysfunction (Hahn et al., 2011; Mora et al., 2012). Several randomized controlled trials have shown that preoperative statin therapy can decrease postoperative atrial fibrillation (AF) (Patti et al., 2006), major adverse cardiovascular and cerebrovascular events (MACCE) after surgery (Xia et al., 2015), myocardial necrosis and short hospital stay (Patti et al., 2006). In addition, some studies have reported that statins involved in neuroprotection (Kivipelto et al., 2005), immunomodulation (Greenwood et al., 2006) and cellular senescence (Brouilette et al., 2007) may reduce the incidence of AKI (Tu et al., 2021). A multicenter prospective cohort study demonstrated that statins might prevent CSA-AKI by detecting the decreased biomarkers of renal injury (Molnar et al., 2014). Layton et al. reported that pre-surgery statin use might reduce the risk of CSA-AKI, especially in younger patients (Layton et al., 2013). Welten et al. found that statin use during admission can improve the recovery of AKI and enhance long-term survival regardless of changes in renal function (Welten et al., 2008). Billings reported that a reduction in CSA-AKI is associated with early postoperative statin use (Billings et al., 2010). Moreover, several studies have demonstrated that in patients with sepsis (Chinaeke et al., 2021) and AKI (Tu et al., 2021), pre-ICU statin use was associated with decreased mortality during the ICU and admission. Although several studies have supported the improvement of statins, others did not (Park et al., 2016; Zheng et al., 2016). Based on these inconsistent conclusions, we intend to further study the relationship between pre-ICU statin use and hospital and 30-day mortality in CSA-AKI patients.

## 2 Materials and methods

### 2.1 Data sources

A retrospective cohort study was conducted using the Medical Information Mart for Intensive Care (MIMIC)-IV version 2.0 (https://physionet.org/content/mimiciv/2.0/), a free and public database. A critical care database for hospitalized patients in Beth Israel Deaconess Medical Center’s ICUs from 2008 to 2019 was included in MIMIC-IV, an update to MIMIC-III. This database was approved by Institutional Review Board. In the Collaborative Institutional Training Initiative, an author has completed the examination (Certification number 42257067 for author Li).

### 2.2 Study population

**FIGURE 1:**
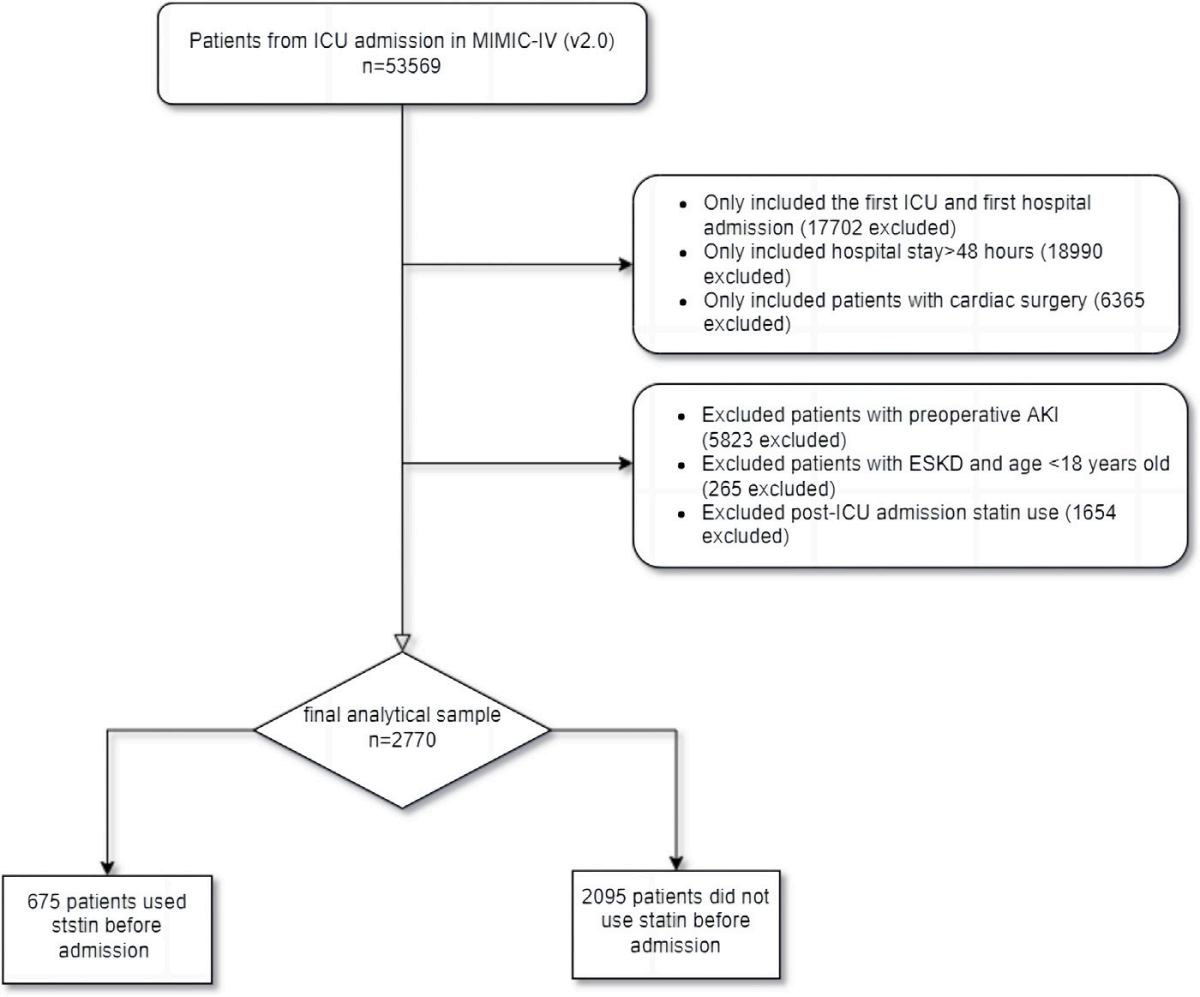
The flowchart of the cohort selection process.

It contains data from 53,569 ICU patients in the database. Patients who met cardiac surgeries during hospitalization according to the ninth and ten revisions of the International Classification of Diseases (ICD-9/10). AKI was defined based on the 2012 KDIGO clinical guidelines: within 7 days, serum creatinine (SCr) increased more than 1.5 times higher than the baseline value (Palevsky et al., 2013). A total of 53,569 patients were admitted to ICU in MIMIC-IV. Patients who met the following criteria were included in the study: 1) first ICU and first hospital admission; 2) hospital stay >48 h; and 3) patients undergoing cardiac surgery during hospitalization and diagnosed with AKI within 1 week after surgery. We excluded patients with preoperative AKI, ESKD, with statins use after admission but without statins before admission (Tu et al., 2021), and aged <18 years. Finally, 675 pre-ICU statin users and 2095 non-users were enrolled in this study (Figure 1), divided into groups pre-ICU statin use and non-use.

### 2.3 Data extraction

Structured Query Language (SQL) was used to extract data in Navicat Premium software (version 15) based on the stay_id and hadm_id of patients. We extract admission information: age, gender, body mass index (BMI), admission type, and AKI stage. The laboratory baseline parameters and vital signs were extracted during the first 24 h of admission to the ICU: white blood cells (WBC), hemoglobin, SCr, bicarbonate, albumin, total cholesterol, respiratory rate, heart rate, oxygen saturation (SpO2), temperature, glucose and mean arterial pressure (MAP). The comorbidities were investigated based on ICD-9/10 codes: hypertension, diabetes, CKD, chronic heart failure (CHF), malignant cancer, and myocardial infarction. In addition, the simplified acute physiology score II (SAPS II), the sequential organ failure assessment (SOFA) score, the charlson comorbidity index (CCI) score, and In-hospital procedures such as hemodialysis and ventilation were collected. Because of many missing total cholesterol data, we referred to other literature to delete it (Tu et al., 2021).

### 2.4 Outcomes

The main outcomes in our study were hospital and 30-day mortality, while the secondary outcomes were 60-day mortality and 90-day mortality.

### 2.5 Statistical analysis

Classification variables were expressed as numbers or percentages. In order to distinguish between the two groups, Mann-Whitney and Chi-squared tests were performed, with a significance level defined as *p* < 0.05. For determining whether the continuous variable has a normal distribution, the Kolmogorov-Smirnov test and Shapiro-Wilk test were used. Continuous variables were shown as median (first quartile, third quartile) because it does not conform to the normal distribution. Variables with less than 40% missing data have been retained (Yang et al., 2020). All missing values were filled with a single interpolation method by SPSS version 25.

In order to reduce the imbalance in baseline covariates between pre-ICU statin use and non-use, an algorithm of 1:1 greedy matching was used in the propensity score matching (PSM) approach, with a 0.01 cutoff-value. The variables selected by PSM were gender, AKI stage, age, admission type, BMI, WBC, hemoglobin, albumin, SCr, bicarbonate, hypertension, diabetes, CKD, CHF, malignant cancer, myocardial infarction, hemodialysis, ventilation, SOFA, SAPS II, CCI, heart rate, MAP, respiratory rate, temperature, SpO2, glucose and ICU LOS. Subsequently, we used Standardized Mean Difference (SMD) to evaluate the similarity of the distribution of covariates in matching samples and compared the outcome before and after PSM.

The Cox regression models for hospital mortality after matching include age, glucose, ventilation, albumin, bicarbonate and statins. And the Cox regression models for 30-day mortality after matching include age, SOFA, CCI, glucose, AKI stage III, ventilation, albumin, SCr, bicarbonate and statins. Meanwhile, we conducted a subgroup analysis to determine whether statin use was associated with CSA-AKI occurrence in patients stratified by age, gender, hypertension, diabetes, CKD, CHF, myocardial infarction, ventilation, SOFA and AKI stage. The Kaplan-Meier analysis was implemented to evaluate the difference in 30-day mortality, 60-day mortality and 90-day mortality between the pre-ICU statin use and the non-use group before and after matching. In order to conduct the above statistical analysis, SPSS version 25 was used. A subgroup forest map is drawn with GraphPad Prism 9.0. Drawing software using Adobe Illustrator 2021.

## 3 Results

### 3.1 Patient selection and baseline characteristics

In this study, 2,770 patients were divided into the pre-ICU statin use group and the non-use group (Figure 1). The significant differences between the two groups were shown before PSM analysis, including age, gender, BMI, admission type, AKI stage, WBC, hemoglobin, albumin, bicarbonate, hypertension, diabetes, CKD, CHF, myocardial infarction, ventilation, hemodialysis, SOFA, SAPS-II, CCI, heart rate, MAP, respiratory rate, temperature and SpO2 (Table 1). Then 423 patients in the pre-ICU statin use group were matched with 423 patients in the non-use group through 1:1 matching. After matching, the differences and imbalances in the baseline characteristics between the two groups can be reduced.

**TABLE 1:** Baseline characteristics before and after propensity-score matching.

	Before matching				After matcing			
	statin prescription				statin prescription			
**Covariate**	User (N = )	Non-users (N = )	ASMD	*p*-value	User (N = )	Non-users (N = )	ASMD	*p*-value
Demographics, n	675	2095			423	423		
Age (years)	74 (67.0, 81.0)	61 (48.0, 74.0)	0.778	<0.001	72 (65.0, 80.0)	73 (63.0, 81.0)	0.016	0.839
Sex, n (%)				0.034				0.78
Female	248 (36.7)	866 (41.3)			178 (42.1)	174 (41.1)		
Male	427 (63.3)	1,229 (58.6)			245 (57.9)	249 (58.9)		
BMI(Mean ± SD)	29.8 (25.8, 34.0)	28.5 (24.4, 33.6)	0.063	0.001	29.1 (25.4, 34.2)	28.7 (24.3, 34.9)	0.007	0.342
Admission type, n (%)				<0.001				0.015
Emergency	157 (23.3)	1,139 (54.3)			118 (27.9)	147 (34.8)		
Elective	92 (13.6)	71 (3.3)			64 (15.1)	37 (8.8)		
Urgent	271 (40.2)	616 (29.4)			138 (32.6)	133 (31.4)		
Other	155 (23)	269 (13)			103 (24.4)	106 (25.1)		
AKI stage, n (%)				<0.001				0.537
I	481 (71.3)	1,131 (53.9)			288 (68.1)	295 (69.7)		
II	115 (17.0)	413 (19.7)			72 (17.0)	76 (18.0)		
III	79 (11.7)	551 (26.3)			63 (14.9)	52 (12.3)		
**Lab variables**								
WBC(K/uL)	11.1 (9.2, 13.0)	11.8 (9.1, 15.0)	0.183	<0.001	11.1 (9.2, 13.3)	11.2 (9.2, 13.1)	0.039	0.998
Hemoglobin (g/dL)	9.8 (9.1, 10.6)	9.6 (8.7, 10.7)	0.065	<0.001	9.9 (9.1, 10.6)	9.9 (8.9, 10.9)	0.049	0.686
Albumin (g/dL)	3.5 (3.1, 3.9)	2.9 (2.5, 3.4)	0.836	<0.001	3.1 (3.0, 3.6)	3.0 (3.0, 3.6)	0.018	0.962
Creatinine (mg/dL)	1.2 (0.9, 1.6)	1.2 (0.8, 2.1)	0.22	0.96	1.2 (0.9, 1.7)	1.1 (0.9, 1.6)	0.011	0.084
Bicarbonate (mEq/L)	25.3 (23.6, 27.0)	24.1 (21.8, 26.5)	0.364	<0.001	25.6 (23.5, 27.9)	25.6 (23.5, 27.9)	0.071	0.146
**Comorbidity, n(%)**								
Hypertension	315 (46.7)	843 (40.2)		0.003	198 (46.8)	201 (47.5)		0.836
Diabetes	323 (47.9)	477 (22.7)		<0.001	161 (38.1)	174 (41.1)		0.361
CKD	221 (32.7)	333 (15.8)		<0.001	118 (27.9)	122 (28.8)		0.76
CHF	127 (18.8)	309 (14.7)		0.012	79 (18.7)	97 (22.9)		0.127
Malignant cancer	29 (4.3)	91 (4.3)		0.958	23 (5.4)	17 (4.0)		0.331
Myocardial infarction	206 (30.5)	102 (4.9)		<0.001	61 (14.4)	56 (13.2)		0.619
**In-hospital procedures, n(%)**								
Ventilation	79 (11.7)	1,019 (48.6)		<0.001	72 (17.0)	86 (20.3)		0.217
Hemodialysis	20 (3.0)	279 (13.3)		<0.001	17 (4.0)	19 (4.5)		0.733
**Laboratory Index**								
SOFA	6 (4.0, 9.0)	8 (5.0, 12.0)	0.488	<0.001	7 (4.0, 9.0)	6 (4.0, 9.0)	0.023	0.601
SAPS II	39 (32.0, 48.0)	41 (32.0, 53.0)	0.124	0.019	40 (33.0, 48.0)	40 (33.0, 48.0)	0.03	0.953
CCI	7 (5.0, 8.0)	5 (3.0, 7.0)	0.575	<0.001	6 (5.0, 8.0)	6 (5.0, 8.0)	0.023	0.821
**Vital signs**								
Heart rate (bpm)	81.3 (74.5, 88.7)	89 (77.5, 102.9)	0.462	<0.001	81.6 (75.1, 89.6)	81.3 (74.6, 91.9)	0.019	0.904
MBP (mmHg)	72.5 (68.2, 76.8)	75.9 (70.1, 83.0)	0.449	<0.001	73.6 (69.3, 78.1)	73.6 (68.3, 78.2)	0.017	0.567
Respiratory rate (bpm)	18.3 (16.6, 20.5)	19.8 (17.3, 23.2)	0.434	<0.001	18.6 (16.8, 20.7)	18.6 (16.6, 21.1)	0.03	0.974
Temperature (°C)	36.7 (36.5, 37.0)	36.9 (36.6, 37.3)	0.203	<0.001	36.8 (36.6, 36.9)	36.8 (36.5, 37.0)	0.021	0.657
SpO2 (%)	97.7 (99.6, 98.4)	97.3 (95.8, 98.6)	0.155	0.007	97.5 (96.1, 98.6)	97.4 (96.2, 98.5)	0.039	0.809
Glucose (mmol/L)	7.4 (6.8, 8.3)	7.6 (6.5, 9.1)	0.069	0.319	7.3 (6.8, 8.3)	7.4 (6.6, 8.8)	0.059	0.688

### 3.2 Outcomes comparisons

Before and after matching, we can see a statistical difference between the two groups. The 30-day mortality, 60-day mortality, 90-day mortality and hospital mortality in the non-use statin group were significantly higher than those in the pre-ICU use statin group (Table 2).

**TABLE 2:** Clinical outcomes before and after propensity-score matching population.

	Before matching			After matching		
Clinical outcomes, (n,%)	Non-users	Users	*p*-value	Non-users	Users	*p*-value
30-day mortality	660 (31.5)	64 (9.4)	<0.001	84 (19.8)	46 (10.8)	<0.001
60-day mortality	750 (35.8)	87 (12.8)	<0.001	99 (23.4)	62 (14.6)	0.001
90-day mortality	801 (38.2)	98 (14.5)	<0.001	111 (26.2)	71 (16.7)	0.001
Hospital mortality	641 (30.6)	62 (9.2)	<0.001	80 (18.9)	47 (11.1)	0.001

### 3.3 Evaluation of risk factors for hospital mortality and 30-day mortality

We will screen statistically significant variables through univariate COX regression analysis and incorporate them into the multivariate COX analysis model. We used multivariate Cox regression analysis to find that age, glucose and ventilation were independent risk factors in-hospital mortality. In contrast, albumin, bicarbonate, pre-ICU statin use (HR = 0.407, 95%CI 0.278–0.595, *p* < 0.001) were an independent protective factor in-hospital mortality (Table 3). The age, SOFA, CCI, glucose, AKI grade III and ventilation were independent risk factors in 30-day mortality. However, albumin, SCr, bicarbonate, pre-ICU statin use (HR = 0.407, 95%CI 0.279–0.595, *p* < 0.001) were an independent protective factor in 30-day mortality (Table 4).

**TABLE 3:** Cox regression analyses to assess risk factors associated with hospital mortality in CSA-AKI patients.

	*p*-Value	HR (95% CI)
Age	0.011	1.026 (1.006–1.046)
Albumin	0.023	1.075 (1.010–1.143)
Bicarbonate	<0.001	0.853 (0.807–0.901)
Glucose	<0.001	1.132 (1.063–1.206)
Ventilation	<0.001	2.162 (1.459–3.204)
Pre-ICU statins use	<0.001	0.407 (0.278–0.595)

**TABLE 4:** Cox regression analyses to assess risk factors associated with 30-day mortality in CSA-AKI patients.

	*p*-Value	HR (95% CI)
Age	0.016	1.023 (1.004–1.043)
Albumin	0.022	0.696 (0.509–0.950)
SCr	0.036	0.802 (0.653–0.986)
Bicarbonate	<0.001	0.842 (0.798–0.888)
SOFA	0.029	1.073 (1.007–1.143)
CCI	0.034	1.083 (1.006–1.166)
Glucose	0.002	1.110 (1.040–1.184)
AKI grade III	0.021	1.764 (1.087–2.862)
Ventilation	0.001	1.900 (1.292–2.795)
Pre-ICU statins use	<0.001	0.407 (0.279–0.595)

### 3.4 Kaplan-Meier analysis

**FIGURE 2:**
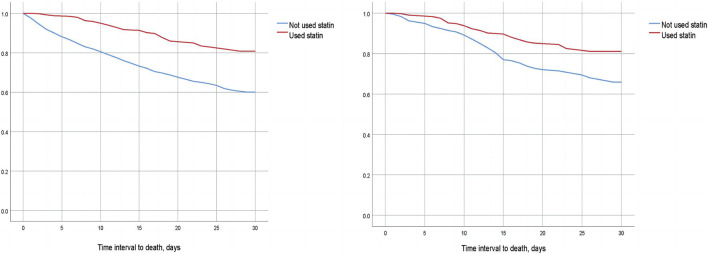
Difference in 30-day mortality between the two groups of CSA-AKI patients before and after matching (Left image: before matching, right image: after matching).

**FIGURE 3:**
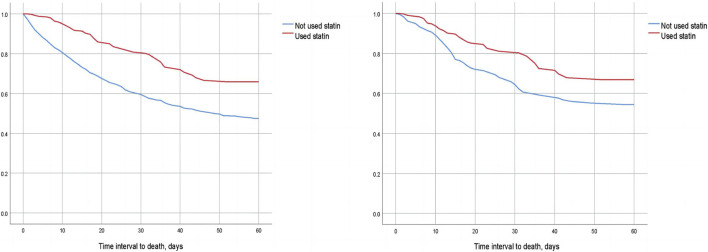
Difference in 60-day mortality between the two groups of CSA-AKI patients before and after matching (Left image: before matching, right image: after matching).

**FIGURE 4:**
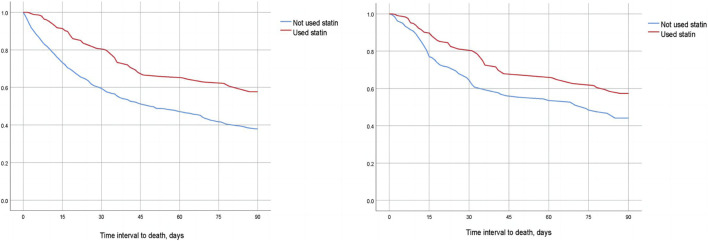
Difference in 90-day mortality between the two groups of CSA-AKI patients before and after matching (Left image: before matching, right image: after matching).

We compared the 30-day, 60-day, and 90-day survival rates of two groups of patients before and after matching through survival analysis. Kaplan-Meier survival analysis showed that the 30-day, 60-day and 90-day survival rates of pre-ICU statin use groups were significantly higher than that of patients in the non-use statin group (log-rank test: *p* < 0.001) (Figure 2; Figure 3; Figure 4).

### 3.5 Subgroup analyses

**FIGURE 5:**
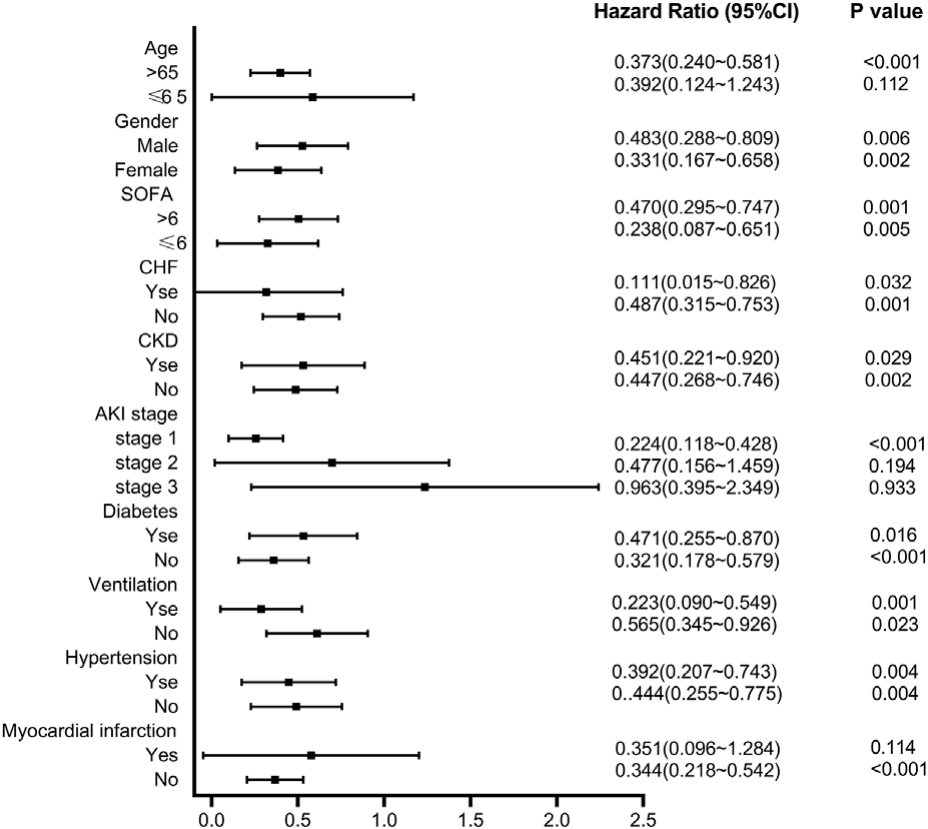
Subgroup analysis of the relationship between pre-ICU statin use and 30-day mortality in CSA-AKI patients.

The subgroup analyses determined the relationship between pre-ICU statin use and 30-day mortality (Figure 5). The result suggested that in subgroups of patients with SOFA≤6, CHF, non-CKD, non-diabetes, ventilation, and hypertension, the 30-day mortality rate of CSA-AKI patients in the statin group was decreased. There was a significant interaction between patients with aged>65 years (HR = 0.373, 95%CI 0.240–0.581, *p* < 0.001), AKI grade I (HR = 0.244, 95%CI 0.118–0.428, *p* < 0.001), and without post-myocardial infarction syndrome (HR = 0.344, 95%CI 0.218–0.542, *p* < 0.001).

### 3.6 Different dose analyses

To further explore the impact of different doses of statins on the mortality rate of CSA-AKI patients, this study specifically focused on the duration of statin use from admission to cardiac surgery. It excluded patients who received multiple doses of statins during this period. Only patients with a single dose record were retained. After screening, it was found that there was a total of 300 people in the statin group. There are 190 patients using atorvastatin, 81 patients using simvastatin, 28 patients using pravastatin, and 1 patient using lovastatin. Considering that the conventional doses used for different statins are different, in this study, we will only discuss the impact of dose differences in atorvastatin, which are the most commonly used and frequently used clinically, in hospital, 30-day, 60-day and 90-day mortality in patients with CSA-AKI. In order to further explore the impact of dose on mortality, patients with CSA-AKI who were treated with atorvastatin ≥80 mg were divided into three groups according to their clinical usage habits, namely,<40 mg, 40–79 mg, and ≥80 mg. As shown in Tables 5 and Tables 8, compared with the non-statin group (using a dose of 0), patients with CSA-AKI who were treated with atorvastatin ≥80 mg had a significant reduction in hospital (*p* = 0.020) and 60-day mortality (*p* = 0.030) (Table 5; Table 6; Table 7; Table 8).

**TABLE 5:** Dose-response relationship between atorvastatin use and hospital mortality in patients with CSA-AKI.

Dose	HR	95%CI	*p*-Value
Non	1		
<40	0.452	0.183–1.117	0.085
40–79	0.537	0.217–1.327	0.178
≥80	0.486	0.264–0.894	0.020

**TABLE 6:** Dose-response relationship between atorvastatin use and 30-day mortality in patients with CSA-AKI.

Dose	HR	95%CI	*p*-Value
Non	1		
<40	0.550	0.240–1.259	0.157
40–79	0.559	0.227–1.379	0.207
≥80	0.558	0.305–1.021	0.059

**TABLE 7:** Dose-response relationship between atorvastatin use and 60-day mortality in patients with CSA-AKI.

Dose	HR	95%CI	*p*-Value
Non	1		
<40	0.481	0.211–1.097	0.082
40–79	1.104	0.592–2.061	0.755
≥80	0.526	0.295–0.938	0.030

**TABLE 8:** Dose-response relationship between atorvastatin use and 90-day mortality in patients with CSA-AKI.

Dose	HR	95%CI	*p*-Value
Non	1		
<40	0.509	0.237–1.094	0.084
40–79	1.101	0.591–2.052	0.762
≥80	0.630	0.378–1.051	0.077

## 4 Discussion

AKI is a major and serious complication after cardiac surgery in connection with increased morbidity and mortality, along with higher medical costs and prolonged hospital stay (Tseng et al., 2020). With the increase in AKI severity, the short-term and long-term mortality rates of CSA-AKI also increase gradually (Hu et al., 2016). Compared with patients without AKI after the operation, the 90-day mortality of CSA-AKI had increased 1.48-fold (Hobson et al., 2015) and the cardiovascular mortality rate at 10-year follow-up also increased (Ozrazgat-Baslanti et al., 2016). In general, cardiac surgery is often associated with renal hypoperfusion due to factors such as low flow, low pressure, hemodilution, and temperature changes (Wang and Bellomo, 2017). Hemorrhagic complications, inflammatory reaction and low cardiac output cause renal ischemia leading to structural renal tubule damage, which may trigger early AKI after surgery (Hudson et al., 2008; Lameire et al., 2008). Some studies demonstrated that cardiopulmonary bypass (CPB), common in cardiac surgery, causes oxidative stress (Baliga et al., 1997) and is associated with activating multiple inflammatory pathways (Bruins et al., 1997). Statins are used to treat hypercholesterolemia, mixed hyperlipidemia, or arteriosclerotic cardiovascular disease (ASCVD), as well as to prevent cardiovascular events. The pleiotropic effect of statins may be beneficial to the cardiovascular system, and this effect is not related to the reduction of low-density lipoprotein. The inhibition of the synthesis of isoprenoid intermediates such as geranylgeranyl pyrophosphate (GGPP) and farnesyl pyrophosphate (FPP) in the cholesterol biosynthetic pathway by statin decreases the post-translational prenylation of small GTP-binding proteins and its downstream effectors (Morofuji et al., 2022). In some studies, there were changes in the production of proinflammatory cytokines and reactive oxygen species, the expression of endothelial nitric oxide synthase, the development of cardiac hypertrophy and fibrosis, the stability of atherosclerotic plaques and the reactivity of platelets (Oesterle et al., 2017; Tian et al., 2017). Besides, statins can be used in CKD patients to reduce cardiovascular risk and delay the progress of CKD (Athyros et al., 2015). Some studies have shown that statins can regulate signal transducer and activator of transcription 6 (STAT6), phosphatase and tensin homolog (PTEN), jumonji domain-containing protein-3 (JMJD3), etc., which are essential in the progression of CKD (An et al., 2022; An et al., 2023). An et al. examined PTEN deficiency exacerbates renal inflammation and fibrosis by regulating the infiltration of macrophages, myeloid fibroblasts, and T lymphocytes into the kidneys (An et al., 2022). Many studies have shown that statins can upregulate the expression of PTEN (Teresi et al., 2006; Teresi et al., 2008; Wu et al., 2013). And Simvastatin can directly inhibit the STAT6 pathway activated by interleukin-13 (IL-13), thereby inhibiting the activation of myeloid fibroblasts and the development of renal fibrosis (Yan et al., 2015; Maneechotesuwan et al., 2016). Statins can promote endothelial cell migration and proliferation by enhancing vascular endothelial growth factor (VEGF) (Matsuno et al., 2004). Recent studies have revealed that VEGF participates in the regulation of renal fibrosis and microvascular sparsity through various mechanisms (Hohenstein et al., 2010; Sison et al., 2010; Basile et al., 2011). These effects have been demonstrated in animal experiments. Kooti et al. showed reduced urinary protein excretion, retained renal function and the elevated mRNA and protein of Adriamycin (ADR)-induced nephropathy rats by pravastatin (Mansouri et al., 2018). Arjinajarn et al. reported that the kidney function of gentamicin-induced rats had been remarkably improved and the inflammation, apoptosis, endoplasmic and reticulum stress were decreased through atorvastatin treatment (Jaikumkao et al., 2016). Fujita et al. demonstrated that ameliorated histopathological damage and urinary protein excretion were decreased in salt-loaded Dahl salt-sensitive rats through the antioxidant as well as depressor effects of pravastatin (Kido et al., 2005). What’s more, statins may also be applied in the treatment of AKI. Statins directly act on endothelial cells by enhancing their ability to generate nitric oxide (NO) and upregulate nitric oxide synthase (eNOS) (Laufs et al., 1997; Kureishi et al., 2000). Research has reported that the use of endothelin receptor antagonists or supplementation with NO can block the enhancement of endothelin and the reduction of endothelial-derived NO release in AKI (Devarajan, 2023).

The role of preoperative statin treatment was controversial in reducing the incidence of CKA-AKI or mortality (Argalious et al., 2010; Park et al., 2016; Zheng et al., 2016). A meta-analysis study suggested statins might reduce the mortality and occurrence of AKI (Li et al., 2018). An observational study showed that preoperative statin therapy has a beneficial influence on mortality (Allou et al., 2010). A retrospective cohort study demonstrated that the risk of early mortality after primary coronary artery bypass graft surgery was reduced by preoperative statin therapy (Pan et al., 2004). Although there was some research on whether pre-ICU statin use can improve the mortality of patients with AKI (Tu et al., 2021) and sepsis (Chinaeke et al., 2021) in the MIMIC database, there is no research on CSA-AKI patients. We mainly studied whether using statins in the pre-ICU is related to the clinical outcome of patients with CSA-AKI and whether it can reduce mortality. Compared with the non-statin use group, we found that the 30-day, 60-day and 90-day mortality was decreased in the pre-ICU statin use group. There was a significant interaction between patients aged>65 years, AKI grade I, and without post-myocardial infarction syndrome in subgroup analysis. The PSM analysis was used to balance the distribution of covariates between the two groups. Our results indicated that statins in the pre-ICU statin use could reduce the mortality of CSA-AKI patients.

There were several limitations in this study. Firstly, we have demonstrated that pre-ICU statins use is associated with the prognosis of patients with CSA-AKI, but we cannot carefully elucidate the specific mechanisms and targets of statins for improving renal function. Secondly, it is a single-center retrospective observational study with small sample size and the MIMIC-IV database including critical patients from 2008 to 2019. There may be inconsistent diagnostic criteria. In future research, we will pay more attention to the type of surgery and statins. And large randomized controlled trials will be conducted to verify the analysis results. The pleiotropic effect of statins also needs to be further studied.

## 5 Conclusion

In conclusion, we confirmed that pre-ICU statin use was significantly associated with decreased risk of hospital and 30-day mortality. The 30-day, 60-day and 90-day survival rates of pre-ICU statin use groups were significantly higher than that of patients in the non-use statin group. However, we need to further explore the prognostic impact of pre-ICU statins use on CSA-AKI patients in the future.

## Data Availability

The original contributions presented in the study are included in the article/supplementary material, further inquiries can be directed to the corresponding author.
